# Recent advances in cancer immunotherapy

**DOI:** 10.1007/s12672-021-00422-9

**Published:** 2021-08-18

**Authors:** Qiang Sun, Gerry Melino, Ivano Amelio, Jingting Jiang, Ying Wang, Yufang Shi

**Affiliations:** 1 Laboratory of Cell Engineering, Institute of Biotechnology, Beijing, China; 2 Research Unit of Cell Death Mechanism, Chinese Academy of Medical Science, Beijing, China; 3 Department of Experimental Medicine, TOR, University of Rome Tor Vergata, 00133 Rome, Italy; 4 DZNE German Center for Neurodegenerative Diseases, Bonn, Germany; 5 School of Life Sciences, University of Nottingham, Nottingham, UK; 6 The Third Affiliated Hospital of Soochow University and State Key Laboratory of Radiation Medicine and Protection, Institutes for Translational Medicine, Soochow University, 199 Renai Road, Suzhou, 215123 Jiangsu China; 7 CAS Key Laboratory of Tissue Microenvironment and Tumor, Shanghai Institute of Nutrition and Health, Chinese Academy of Sciences, 320 Yueyang Road, Shanghai, 200031 China

**Keywords:** Cancer immunotherapy, Immune check point blockade, T cell receptor, Cancer survival, p53, KRas

## Abstract

Cancer immunotherapy represents a major advance in the cure of cancer following the dramatic advancements in the development and refinement of chemotherapies and radiotherapies. In the recent decades, together with the development of early diagnostic techniques, immunotherapy has significantly contributed to improving the survival of cancer patients. The immune-checkpoint blockade agents have been proven effective in a significant fraction of standard therapy refractory patients. Importantly, recent advances are providing alternative immunotherapeutic tools that could help overcome their limitations. In this mini review, we provide an overview on the main steps of the discovery of classic immune-checkpoint blockade agents and summarise the most recent development of novel immunotherapeutic strategies, such as tumour antigens, bispecific antibodies and TCR-engineered T cells.

## Introduction

The mechanism of action of cancer immunotherapy strategies is to activate the immune system against target antigens that are selectively expressed in malignant cells but not in cells of normal tissues. With the Nobel Prize in Physiology or Medicine 2018, awarded jointly to James P. Allison and Tasuku Honjo "*for their discovery of cancer therapy by inhibition of negative immune regulation,*" cancer immunotherapy entered a new phase of exploration. We can identify some key steps that brought us to this therapeutical revolution.

Cancer immunotherapy was jump-started by Tak Mak’s seminal publications [1, 2] describing the cloning of the gene encoding the beta-chain of the human T-cell-receptor (TCR), considered at the time to be the “*Holy Grail of Immunology.*” This was back-to-back with the independent cloning of the mouse equivalent gene by Mark Davis [3, 4]. They followed their remarkable discovery with incredible additional outstanding studies further expanding the topic with the structure of the TCR-encoding genes in human and mouse, the diversity elements within these genes, the expression patterns of TCR transcripts in various T-cell subsets, and the rearrangement of TCR genes in T-cell leukaemia lines [5–8].

The value of this pioneering work is now becoming more and more evident with the importance of the immune system in the fight against cancer with another T-cell receptor activation related molecule, CTLA4 [9], as recognized and highlighted by the work of James Allison [10]. Indeed, following the discovery of TCR, all efforts went into the identification of many more receptors on T lymphocytes and also the understanding of their function in physiological immune responses and in particular those against cancer cells. One in particular is the CTLA4 receptor, which is a negative regulator of T-cell function, acts in concert with CD28 to fine-tune a set of important pathophysiological processes, including chronic autoimmune inflammation, interaction with dendritic cells (DCs) and other antigen presenting cells, stimulation and expansion of the immune response. Mak was the first to show in genetically modified mice that lacking CTLA-4 resulted in a progressive accumulation of T cell blasts, demonstrating that it is a negative regulator of immune responses [9]. CTLA-4 knockout mice have super-activated and proliferating T-cells, demonstrating how important this receptor is in regulating T-cell responses. This became the scientific base on which Allison, by blocking CTLA-4, showed a dramatic anti-tumour efficacy.

Allison explored whether CTLA-4 blockade could be instrumental to disengage the brake on T-cell activation and unleash the immune system to recognise and target malignant cells. Allison with his team initiated to approach this strategy at the end of 1994: they achieved therapeutic response in pre-clinical models of mouse cancers by treatment with the first immune checkpoint blockade agent (*i.e*. antibodies against CTLA-4) [10]. This finding paved the way for utilizing agents that block immune checkpoints, such as PD-1 and PD-L1, as a treatment for oncological patients. Clinical development ensued in the recent years and since 2012 several key studies demonstrated unquestionable efficacy in the treatment of patients with different cancer types [11].

In 1992, Tasuku Honjo discovered another T-cells surface protein, PD-1, based on activation induced cell death in T cells [12, 13] and growth factor deprivation-induced cell death [14]. Similar to CTLA-4, PD-1 was proven to function as a T-cell activation brake but operating with a different mechanism [15]. Now, additional T cell receptor activation regulators have been identified, like PD1, and by eliminating these regulation (blocking CTLA4, PD-1, PD-L1) incredible therapeutic effects have been uncovered. Indeed, clinical application based on these concepts is currently the most efficient way to treat several human cancers that were otherwise untreatable.

Immunotherapy is now our most promising and powerful weapon in combatting malignancies, and the clinical applications of immunology would not have been possible without findings by Mak, Davis and Allison. With the introduction of immune checkpoint blockade (ICB), manipulation of autologous anti-tumour response has become an effective strategy to cure cancer [16]. Despite the significant impact that ICB has provided on overall cancer prognosis, however, a substantial fraction of patients does not display response to the treatment. Except possessing a reasonably intact immune system to allow ICB to release the immunity, a better molecular and functional characterisation of tumour neoantigens has enabled the next generation of cancer immunotherapies [17–22].

Nonetheless, since the Nobel Prize to James P. Allison and Tasuku Honjo, cancer immunotherapy has moved forward rapidly. Two recent collaborative studies simultaneously published by B. Volgelstein, SB. Gabelli and S. Zhou groups describe the development of bispecific antibodies selectively targeting different tumour specific antigens, such as p53 mutant R175H [23] and RAS mutants G12V, Q61H/RL [24]. This work has been complemented by a third article from the same teams, where the authors with a similar approach show the development of bispecific antibodies selectively targeting a T cell-associated TCRβ to treat T cell malignancies [25]. These studies generate important expectation in the next generation of immuno-therapeutics.

## Bispecific antibodies as an innovative cancer immunotherapy

**Fig. 1:**
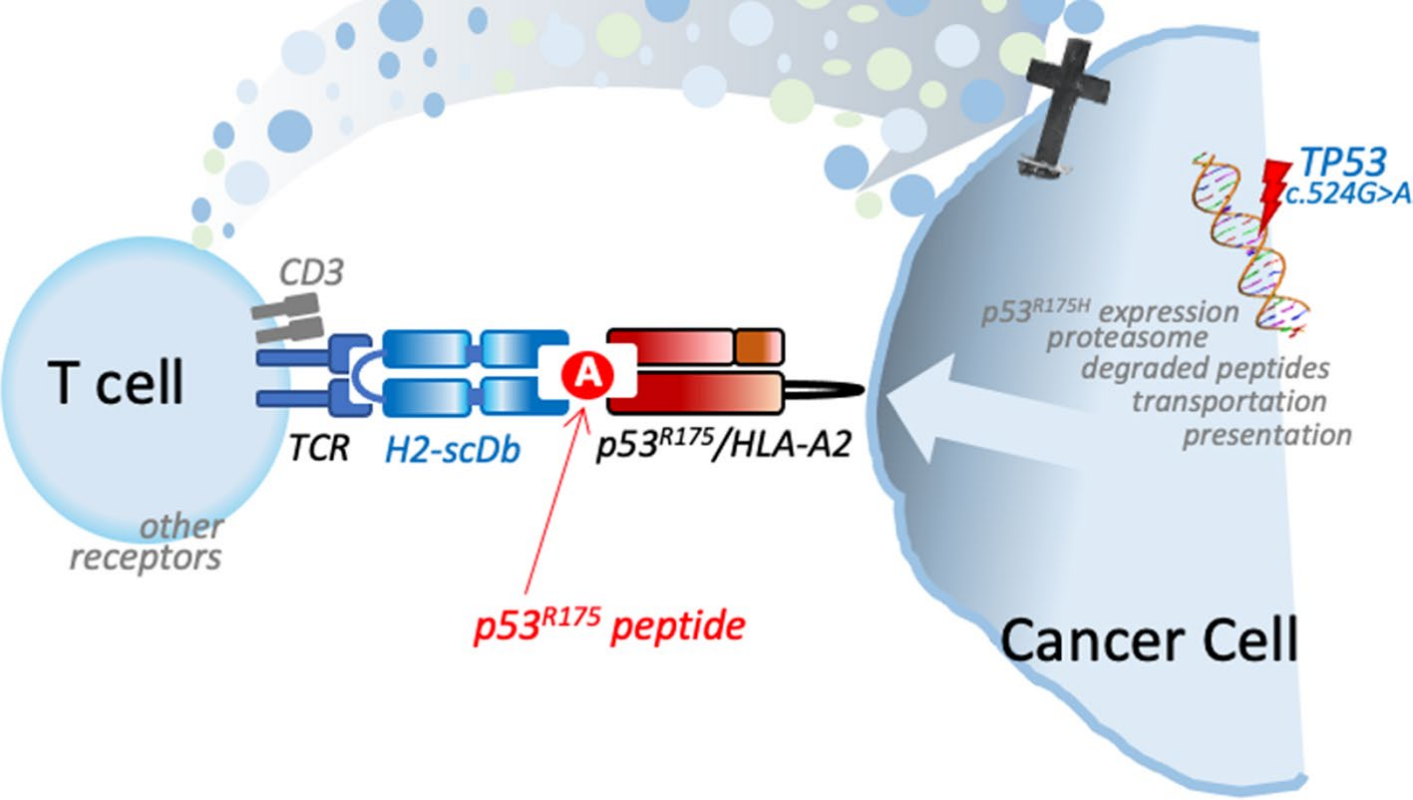
Schematic representation of the mechanism of action of scDb against mutant p53. Tumour cells as able to express on their surface specific peptides, including the R175H mutation of p53, presented by the HLA complex. The Bi-Specific Ab bridging T cells and tumour cell, H2-scDb (blue), includes a TCR arm as well as a p53R175 peptide complex arm. This binds via the p53R175 peptide (red), the peptide presenting complex (dark red/black). Therefore, the tumour cells to the T-cells can be bridged the HLA complex, and on the other arm the CD3/TCR complex from the T lymphocytes. Activation of the T-cell results in killing of the cancer cells, mediated by the release of cytotoxic molecules; in particular via the local cytokine storm includes granzymes, perforin, TNF-a as well as IFN-g, able to activate the programmed cell death program of the cancer cell. The effect of granzymes are mostly exerted through cell–cell contact. scDb, single-chain diabody; HLA, human leukocyte antigen; TCR, T cell Receptor; CD3, cluster of differentiation 3; IFNγ, Interferon gamma; TNF-α, Tumour Necrosis Factor alfa

The process of antigen processing and presentation is mediated by the major histocompatibility complex (MHC) class I and class II antigens and is central to the development, survival, activation and tolerance of the adaptive immune system. The human MHC locus on chromosome 6 included a significant number of polymorphic antigen-presenting genes, each has several haplotypes, that constitute to the variation of the human leukocyte antigen (HLA) class I and II molecules. Novel peptides (neo-peptides) generated by accumulation of mutations during tumorigenesis, are loaded onto HLA class I molecules and presented on the external cell membrane of tumour cells [17]. These neoantigenes can also be cross-presented by professional antigen presenting cells. Whilst tumour antigens can still be classified as tumour-associated antigens (TAAs) or tumour-specific antigens (TSAs), the former are aberrantly expressed by cancer cells [26, 27], but still expressed in normal cells, hence they are not tumour specific. On the other hand, TSAs can be detected exclusively on cancer cells because they are generated by genetic somatic mutations. Recent studies reported bispecific antibodies constituted by a fragment with high affinity for the mutant peptide-HLA complex on the cancer cells, but not for its wild-type counterpart exposed on normal tissue cells, fused to a second antibody fragment that binds to the T cell receptor–CD3 complex on T cell. This agent effectively activates T cells to secrete cytokines and kill target cancer cells (Fig. 1). This strategy in fact overcomes the challenge of directly targeting intracellular proteins such as p53 and RAS, which fall within the most mutate genes across cancers [28–30], while at the same time exploits autologous anti-tumour response to kill the tumour.

In fact, p53 is an excellent target as public neoantigen, considering that it is by far the most frequently mutated gene [31–34] and it has roles in regulating crucial factors in cancer biology such as cell death [35–39], cell cycle [40], cell metabolism [41–48], as well as commensal microbes [20, 49–51]. This is in keeping the function of the other members of the same family, namely p63 and p73 [52–59]; and with the ability to predict overall cancer  patient  survival [60–62]as other prognostic factors in cancer progression [27, 63, 64]. TP53 has several hotspot mutations, and within these, the most frequent mutation is the Arg175His, which has been effectively targeted with bispecific antibodies [30].

**Fig. 2:**
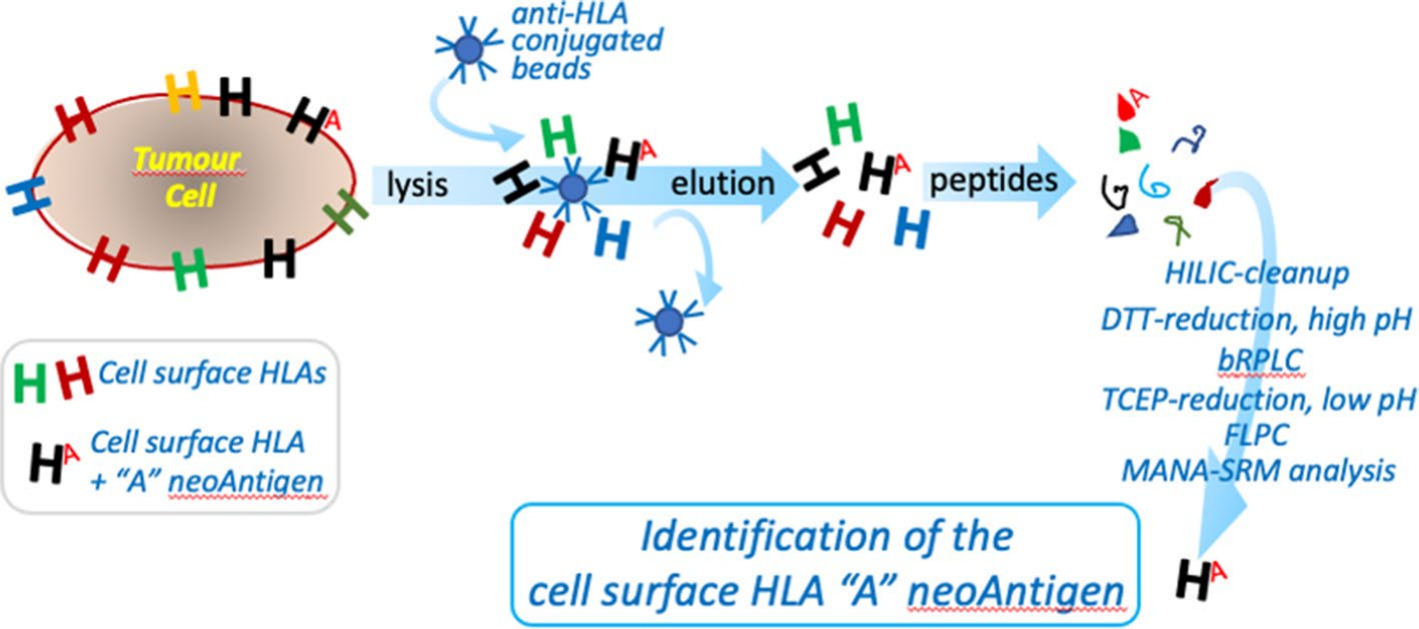
Purification and identification of the cell surface HLA"A" neo-antigen against p53 R175H. The Mutation-Associated Neoantigen—Selected Reaction Monitoring (MANA-SRM) pipeline starts by lysing the cells, and enriching HLA-bound peptides through immuno-precipitation with an antibldy targeting HLA molecules. HLA molecules together with their presented peptides are then eluted and dissociated. At this stage, the eluate containing the neo-antigenic peptides is filtred for lower molecular weights using a cut off of 3 kDa and the samples are lyophilized and analyzed for MANA-SRM using an improved hydrophilic interaction liquid chromatography (HILIC) cleanup; reduction at different pH using DL-dithiothreitol (DTT) or tris(2-carboxyethyl)phosphine (TCEP) is used before analysing the optimization of collision energies and fractionation. Manually examination and curation to exclude ions with excessive noise due to coelution with impurities is required to detect heavy isotope-labeled neo-antigens. See the original reference for details (Wang et al. Cancer Immunol Res, 2019)

To identify and characterize these new antigens on cancer cells, originated by the arginine to histidine substitution on the residue 175 (R175H) in the *TP53* gene [32], Hsiue and colleagues performed a positive selection phage display using naïve human antibody specific to HLA libraries, namely HLA-A*02:01 pHLA monomers, which loaded with the p53 R175H peptide, combined with negative selection against pHLA monomers loaded with the p53 wild type peptide. Figure 2 provides some additional details on the complex procedure to generate antibodies specific to the neo-antigens. The phage clones selected with this procedure were then used to develop the bispecific antibody. This was generated by joining in a single-chain diabody (scDb) each individual scFv to an anti-CD3e scFv (UCHT1); this latter conferred binding ability to the CD3 expsed on T cells, thus promoting a polyclonal T cell response. The specificity of the scDbs for p53 R175H ranked at high affinity was validated with a set of functional assays verifying its inability to target cell line not expressing p53 R175H and cell lines losing expression of p53 R175H following CRISPR-mediated deletion; this procedure was able to sharply reduce the risk of cross-reactivity and therefore of wanton off-target effects. Classic structural biology studies as well as cell biochemical analyses of the scDb binding to mutated p53R175H/HLA-A*02:01 demonstrated the binding specificity of scDbs. Indeed, H2-scDb was able to specifically eliminate p53 R175H tumour cells both in vitro and in vivo in a manner that is strictly dependent on T-cells. As a control for the latter, the authors reported the absence of effects of H2-scDb in NOD-SCID-Il2rg^−/−^ mice engrafted with tumor cells  expressing p53 R175H, without simultaneous engraftment with human T cells [23].

Interestingly, the authors have used a comparable approach using the RAS G12V and Q61H/L/R mutations in cancer cells. This is important, as it demonstrate the general validity of the use of scDbs targeting HLA alleles conjugated to a different set of cancer mutations. In this latter case, Douglass and colleagues identified and characterized a highly specific scDbs directed against mutant RAS; here too, the effect on tumour cells expressing very low HLA copies was evident and powerful [24]. A general remark however remains the possibility of cross-reactivity to other human peptides when administrated in patients. Safety related to off targets effects remains to be assessed in patients.

An innovative scientific strategy was at the bases of the biochemical purification, isolation and characterization of scDbs specifically directed against T-cell cancers. Indeed, the TCR β-chain variable regions includes any of the circa 30 possible TRBV family members; now, if we consider that T cell malignancies are clonal, only a single TRBVcan be exposed on the external cell membrane of the malignant T cells. Accordingly, TCR β-chain of an individual malignant T cell is a perfect TAA. In line with this, Paul et al. generated and characterized specific scDbs directed toward TRBV5-5 or TRBV12, tethered to a CD3 antibody. These specific scDbs are able to specifically kill cancerous T cells transplanted in humanized mice, preserving the majority of healthy human T cells not expressing the targeted TRBV. In their case, Paul et al. did not require a complex phage display identification of the targeting antibody, as they were able to, more simply, use antibodies specific to the TCR Vβs, TRBV5-5 and TRBV12 as a proof of principle [25]. Indeed, whilst less innovative, bispecific antibodies have already been developed to target aberrantly expressed, not mutated, cancer proteins (TAAs) [65].

Targeting TSAs, generated by mutated oncogenes/oncosuppressors, with bispecific antibodies appears particularly innovative despite the fact that the concept has been on the road for quite a few years; TSAs presented by HLAs have been targeted only by adaptive T cell therapies [66, 67]. However, a potential problem of this more classical therapy, as compared to scDbs, is that the adaptive T cell procedure requires extensive, expensive and complex experimental approach with sophisticated ex vivo manipulations for individual patient's autologous immune cells [68]. On the other hand, scDbs may be an off-shelf easily accessible medicament with wide therapeutic potentials. The peptide derived from the p53 R175H, for examples, binds the HLA-A*02:01 that is present in almost half of the Caucasian population in the United States, thus the developed H2-scDb could be engaged to treat a substantial fraction of cancer patients.

Remarkable significance of these studies lies in the formal demonstrations of the possibility to develop agents with very high affinity (recognitions of very low HLA expressing cancer cells), with very high specificity (mutant vs wt HLAs), shifting the emphasis of the novel approaches in cancer immunotherapy from the cell-based strategies to bifunctional antibodies. The route to clinics appears, however, still ambitious, as several issues remain to be evolving. Many tumours, for examples, deploy a massive immunosuppressive repertoire of signalling molecules and how scDbs will function in this landscape has to be verified [69]. Moreover, a large fraction of immunotolerant cancers downregulate HLA as a major mechanism of immune evasion and these will remain out of the radar of this approach [70]. Complex integration of environments, life-style, and dietary factors could further influence the immune system, via metabolic regulations [48, 61, 69, 71–76], epigenetics [77–80], autophagy [81–86], interaction with hormone signalling [87–92] and microbiome [50, 93–95], thus altering the response to these categories of immunotherapeutic agents. Additional clarifications are demanded on the antigenicity of scDB to induce idiotypic responses and more in general on their safety on patients. Is the specificity of the antibody sufficient to preserve safety, but conserve therapeutic efficacy across different individuals carrying the same mutations? Not last, whether the immune response elicited by the antibody is sufficient to eliminate cancerous cells in a complex metastatic clinical setting where the ratio of T cell:cancer cells is rather variable represents a major question to be addressed.

## TCR-engineered T cells

More innovative genetic manipulation of the TCR, beside the already discussed chimeric antigen receptor redirected T cells(CAR-T) approach, originally defined by Zelig Eshhar [96, 97] (and reviewed in D'Aloia et al. [98]; Melero et al. [99]; Morris et al. [100]), are now emerging to be effective in different cancer models. CARs are transmembrane chimeric molecules acting as: (a) immune recognition of neoplastic new antigens present on the cell surface of cancers; (b) promotion and propagation of molecular events activating the cell lysis. CAR-T leads to: (1) “reprogrammed T-cells” with an ex-novo activation mechanism; (2) break the tolerance generated in the cancer microenvironment, and (3) bypass restrictions of the HLA-mediated antigen recognition, over-stepping one of the barriers to a more widespread application of cellular immunotherapy. Even tough applied to selective patients, this approach is now clinically effective [71, 101].

**Fig. 3:**
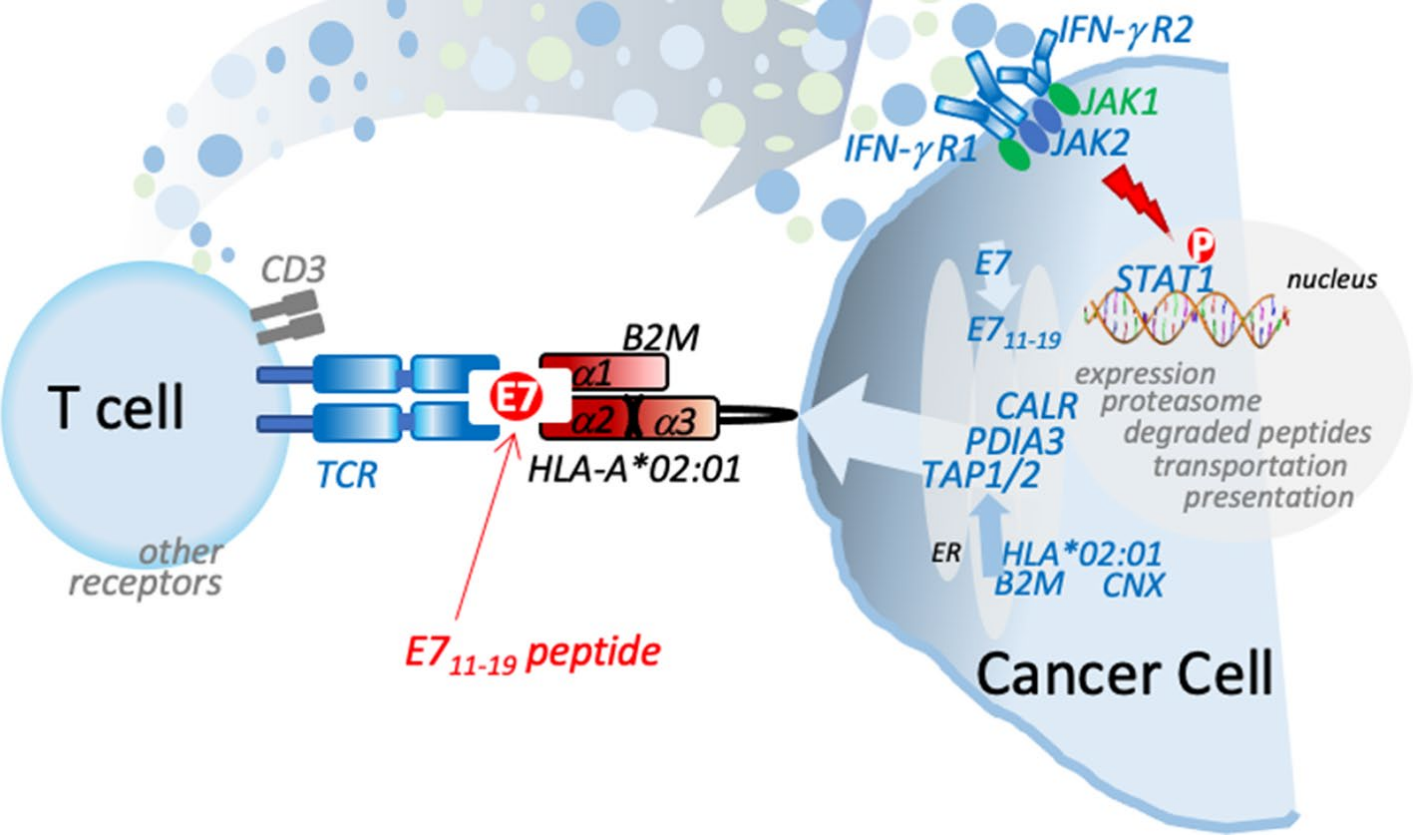
Schematic representation of the HPV-16 E7 specific TCR engineered T cell for patients with metastatic HPV-associated epithelial cancers. Simplified scheme showing the E711-19 specific TCR in a patient with a defect in the antigen processing is able to trigger a IFN response. A high-avidity TCR that targets HPV-16 E7 through the recognition of the E711–19 epitope complexed with HLA-A*02:01.Genetically engineered T cells are able to engage and kill HPV + tumour cell lines in vitro and mediate regression of HPV + tumor xenografts in vivo as well as in human patients. See for more details (*Nagarsheth, Nat Med-2021*[102]*). Since the CD3 specific arm could activate both CD4 and CD8 cells, the cancer cell killing mechanism could be much more than IFNγ. In addition, the ability of IFNγ to induce HLA expression could be very important*

Recently, high-avidity TCR that targets HPV-16 E7 through recognition of the E711–19 epitope complexed with HLA-A*02:01 was reported [102]. Here, engineered T cell receptor targeting HPV-16 E7 for the treatment of metastatic human papillomavirus-associated epithelial cancers were administered up to10^11^ cells. The primary tumours were squamous cell carcinomas from head and neck, cervical, or anal primary sites. Figure 3 shows the rationale of the approach, based on an engineered TCR recognising HLA-A*02:01 conjugated to E7 peptide, which triggers a cytokine release able to kill the cancer cell. Significant regression was observed in 12 patients, including those with anti-PD-1 refractory disease.

While there are a number of distinct HLA, each one displaying different peptides with different affinity, current cancer immunotherapy can only target a limited set of HLA isoforms, severely restricting its accessibility to patients. More recently, Hirano and Mak have developed a new strategy that they validated in 8 patients with melanoma [103]. Generating a library of peptide exchangable HLA-peptide multimers to activate Tumour Infiltrating Lymphocytes (TIL). With 25 forms of HLA to display over 800 peptides, the authors were able to select A*02:01/MART1_27-35_ as a relevant immunological hotspot, with the successful staining up to 9.2% of the polyclonally expanded TILs. Using this rationale [104], they further identified shared antigenic epitopes recognized by melanoma TILs and their cloned TCRs, for the development of novel cancer immunotherapy [103]. This, certainly changes the landscape of tumour immunotherapy [105].

In the last decade, the molecular definition of TCR specificity through peptide-bounding multimers of MHC molecules has become available approachs to target cancer specifically. This will certainly impact the current prognostic landscape [51, 106–108]. Despite the high cost associated with T-cell based therapies, the flexibility of this approach still makes it a particularly attractive strategy to cure cancer. The implementation of modern genetic manipulation techniques, such as CRISPR/Cas9, has further expanded the repertoire of applications. A general consensus has been achieved that manipulation of immune system is the avenue to undertake to develop novel cancer therapies that provide efficacy, effectiveness and wider applications across different tumour types.

## Data Availability

Not applicable.

## References

[1] Yanagi Y, Yoshikai Y, Leggett K, Clark SP, Aleksander I, Mak TW (1984). A human T cell-specific cDNA clone encodes a protein having extensive homology to immunoglobulin chains. Nature.

[2] Mak TW (2021). From the T-cell receptor to cancer therapy: an interview with Tak W. Mak Cell Death Differ.

[3] Hedrick SM, Nielsen EA, Kavaler J, Cohen DI, Davis MM (1984). Sequence relationships between putative T-cell receptor polypeptides and immunoglobulins. Nature.

[4] Hedrick SM, Cohen DI, Nielsen EA, Davis MM (1984). Isolation of cDNA clones encoding T cell-specific membrane-associated proteins. Nature.

[5] Mak TW (2007). The T cell antigen receptor: "The Hunting of the Snark". Eur J Immunol.

[6] Davis MM, Boniface JJ, Reich Z, Lyons D, Hampl J, Arden B, Chien Y (1998). Ligand recognition by alpha beta T cell receptors. Annu Rev Immunol.

[7] Newell EW, Davis MM (2014). Beyond model antigens: high-dimensional methods for the analysis of antigen-specific T cells. Nat Biotechnol.

[8] Toyonaga B, Mak TW (1987). Genes of the T-cell antigen receptor in normal and malignant T cells. Annu Rev Immunol.

[9] Waterhouse P, Penninger JM, Timms E, Wakeham A, Shahinian A, Lee KP, Thompson CB, Griesser H, Mak TW (1995). Lymphoproliferative disorders with early lethality in mice deficient in Ctla-4. Science.

[10] Leach DR, Krummel MF, Allison JP (1996). Enhancement of antitumor immunity by CTLA-4 blockade. Science.

[11] Chen DS, Mellman I (2017). Elements of cancer immunity and the cancer-immune set point. Nature.

[12] Shi YF, Sahai BM, Green DR (1989). Cyclosporin A inhibits activation-induced cell death in T-cell hybridomas and thymocytes. Nature.

[13] Ashwell JD, Cunningham RE, Noguchi PD, Hernandez D (1987). Cell growth cycle block of T cell hybridomas upon activation with antigen. J Exp Med.

[14] Palacios R, Karasuyama H, Rolink A (1987). Ly1+ PRO-B lymphocyte clones. Phenotype, growth requirements and differentiation in vitro and in vivo. EMBO J.

[15] Ishida Y, Agata Y, Shibahara K, Honjo T (1992). Induced expression of PD-1, a novel member of the immunoglobulin gene superfamily, upon programmed cell death. EMBO J.

[16] Topalian SL, Taube JM, Anders RA, Pardoll DM (2016). Mechanism-driven biomarkers to guide immune checkpoint blockade in cancer therapy. Nat Rev Cancer.

[17] Gupta RG, Li F, Roszik J, Lizee G (2021). Exploiting tumor neoantigens to target cancer evolution: current challenges and promising therapeutic approaches. Cancer Discov.

[18] Messmer MN, Snyder AG, Oberst A (2019). Comparing the effects of different cell death programs in tumor progression and immunotherapy. Cell Death Differ.

[19] Oktay K, Santaliz-Casiano A, Patel M, Marino N, Storniolo AMV, Torun H, Acar B, Madak Erdogan Z (2020). A computational statistics approach to evaluate blood biomarkers for breast cancer risk stratification. Horm Cancer.

[20] Celardo I, Melino G, Amelio I (2020). Commensal microbes and p53 in cancer progression. Biol Direct.

[21] Larmuseau M, Verbeke LPC, Marchal K (2019). Associating expression and genomic data using co-occurrence measures. Biol Direct.

[22] Khairi S, Osborne J, Jacobs MF, Clines GT, Miller BS, Hughes DT, Else T (2020). Outcome of clinical genetic testing in patients with features suggestive for hereditary predisposition to PTH-mediated hypercalcemia. Horm Cancer.

[23] Hsiue EH, Wright KM, Douglass J, Hwang MS, Mog BJ, Pearlman AH, Paul S, DiNapoli SR, Konig MF, Wang Q (2021). Targeting a neoantigen derived from a common TP53 mutation. Science.

[24] Douglass J, Hsiue EH, Mog BJ, Hwang MS, DiNapoli SR, Pearlman AH, Miller MS, Wright KM, Azurmendi PA, Wang Q (2021). Bispecific antibodies targeting mutant RAS neoantigens. Sci Immunol.

[25] Paul S, Pearlman AH, Douglass J, Mog BJ, Hsiue EH, Hwang MS, DiNapoli SR, Konig MF, Brown PA, Wright KM (2021). TCR beta chain-directed bispecific antibodies for the treatment of T cell cancers. Sci Transl Med.

[26] Panchin AY, Aleoshin VV, Panchin YV (2019). From tumors to species: a SCANDAL hypothesis. Biol Direct.

[27] Mihaylov I, Kandula M, Krachunov M, Vassilev D (2019). A novel framework for horizontal and vertical data integration in cancer studies with application to survival time prediction models. Biol Direct.

[28] Levine AJ (2020). p53: 800 million years of evolution and 40 years of discovery. Nat Rev Cancer.

[29] Drosten M, Barbacid M (2020). Targeting the MAPK Pathway in KRAS-Driven Tumors. Cancer Cell.

[30] Mantovani F, Collavin L, Del Sal G (2019). Mutant p53 as a guardian of the cancer cell. Cell Death Differ.

[31] Ham SW, Jeon HY, Jin X, Kim EJ, Kim JK, Shin YJ, Lee Y, Kim SH, Lee SY, Seo S (2019). TP53 gain-of-function mutation promotes inflammation in glioblastoma. Cell Death Differ.

[32] Pitolli C, Wang Y, Mancini M, Shi Y, Melino G, Amelio I (2019). Do Mutations Turn p53 into an Oncogene?. Int J Mol Sci.

[33] Pitolli C, Wang Y, Candi E, Shi Y, Melino G, Amelio I (2019). p53-mediated tumor suppression: DNA-damage response and alternative mechanisms. Cancers (Basel).

[34] Amelio I, Mancini M, Petrova V, Cairns RA, Vikhreva P, Nicolai S, Marini A, Antonov AA, Le Quesne J, Baena Acevedo JD (2018). p53 mutants cooperate with HIF-1 in transcriptional regulation of extracellular matrix components to promote tumor progression. Proc Natl Acad Sci USA.

[35] Kim SY, Nair DM, Romero M, Serna VA, Koleske AJ, Woodruff TK, Kurita T (2019). Transient inhibition of p53 homologs protects ovarian function from two distinct apoptotic pathways triggered by anticancer therapies. Cell Death Differ.

[36] Li Y, Cao Y, Xiao J, Shang J, Tan Q, Ping F, Huang W, Wu F, Zhang H, Zhang X (2020). Inhibitor of apoptosis-stimulating protein of p53 inhibits ferroptosis and alleviates intestinal ischemia/reperfusion-induced acute lung injury. Cell Death Differ.

[37] Li X, Guo M, Cai L, Du T, Liu Y, Ding HF, Wang H, Zhang J, Chen X, Yan C (2020). Competitive ubiquitination activates the tumor suppressor p53. Cell Death Differ.

[38] Liu H, Weng W, Guo R, Zhou J, Xue J, Zhong S, Cheng J, Zhu MX, Pan SJ, Li Y (2020). Olig2 SUMOylation protects against genotoxic damage response by antagonizing p53 gene targeting. Cell Death Differ.

[39] Liang J, Niu Z, Zhang B, Yu X, Zheng Y, Wang C, Ren H, Wang M, Ruan B, Qin H (2021). p53-dependent elimination of aneuploid mitotic offspring by entosis. Cell Death Differ.

[40] Huang S, Li Y, Yuan X, Zhao M, Wang J, Li Y, Li Y, Lin H, Zhang Q, Wang W (2019). The UbL-UBA Ubiquilin4 protein functions as a tumor suppressor in gastric cancer by p53-dependent and p53-independent regulation of p21. Cell Death Differ.

[41] Lonetto G, Koifman G, Silberman A, Attery A, Solomon H, Levin-Zaidman S, Goldfinger N, Porat Z, Erez A, Rotter V (2019). Mutant p53-dependent mitochondrial metabolic alterations in a mesenchymal stem cell-based model of progressive malignancy. Cell Death Differ.

[42] Espinoza JA, Zisi A, Kanellis DC, Carreras-Puigvert J, Henriksson M, Huhn D, Watanabe K, Helleday T, Lindstrom MS, Bartek J (2020). The antimalarial drug amodiaquine stabilizes p53 through ribosome biogenesis stress, independently of its autophagy-inhibitory activity. Cell Death Differ.

[43] Nepravishta R, Sabelli R, Iorio E, Micheli L, Paci M, Melino S (2012). Oxidative species and S-glutathionyl conjugates in the apoptosis induction by allyl thiosulfate. FEBS J.

[44] Angelucci S, Sacchetta P, Moio P, Melino S, Petruzzelli R, Gervasi P, Di Ilio C (2000). Purification and characterization of glutathione transferases from the sea bass (Dicentrarchus labrax) liver. Arch Biochem Biophys.

[45] Pallucca R, Visconti S, Camoni L, Cesareni G, Melino S, Panni S, Torreri P, Aducci P (2014). Specificity of epsilon and non-epsilon isoforms of arabidopsis 14–3–3 proteins towards the H+-ATPase and other targets. PLoS ONE.

[46] Mauretti A, Neri A, Kossover O, Seliktar D, Nardo PD, Melino S (2016). Design of a novel composite H2 S-releasing hydrogel for cardiac tissue repair. Macromol Biosci.

[47] Amelio I, Melino G (2015). The p53 family and the hypoxia-inducible factors (HIFs): determinants of cancer progression. Trends Biochem Sci.

[48] Dobon B, Montanucci L, Pereto J, Bertranpetit J, Laayouni H (2019). Gene connectivity and enzyme evolution in the human metabolic network. Biol Direct.

[49] Kawulok J, Kawulok M, Deorowicz S (2019). Environmental metagenome classification for constructing a microbiome fingerprint. Biol Direct.

[50] Caputo A, Fournier PE, Raoult D (2019). Genome and pan-genome analysis to classify emerging bacteria. Biol Direct.

[51] Walker AR, Datta S (2019). Identification of city specific important bacterial signature for the MetaSUB CAMDA challenge microbiome data. Biol Direct.

[52] Bellomaria A, Barbato G, Melino G, Paci M, Melino S (2010). Recognition of p63 by the E3 ligase ITCH: effect of an ectodermal dysplasia mutant. Cell Cycle.

[53] Lena AM, Cipollone R, Amelio I, Catani MV, Ramadan S, Browne G, Melino G, Candi E (2010). Skn-1a/Oct-11 and DeltaNp63alpha exert antagonizing effects on human keratin expression. Biochem Biophys Res Commun.

[54] Nemajerova A, Amelio I, Gebel J, Dotsch V, Melino G, Moll UM (2018). Non-oncogenic roles of TAp73: from multiciliogenesis to metabolism. Cell Death Differ.

[55] Amelio I, Antonov AA, Catani MV, Massoud R, Bernassola F, Knight RA, Melino G, Rufini A (2014). TAp73 promotes anabolism. Oncotarget.

[56] Amelio I, Cutruzzola F, Antonov A, Agostini M, Melino G (2014). Serine and glycine metabolism in cancer. Trends Biochem Sci.

[57] Bellomaria A, Barbato G, Melino G, Paci M, Melino S (2012). Recognition mechanism of p63 by the E3 ligase Itch: novel strategy in the study and inhibition of this interaction. Cell Cycle.

[58] Vikhreva P, Melino G, Amelio I (2018). p73 Alternative Splicing: Exploring a Biological Role for the C-Terminal Isoforms. J Mol Biol.

[59] Grespi F, Amelio I, Tucci P, Annicchiarico-Petruzzelli M, Melino G (2012). Tissue-specific expression of p73 C-terminal isoforms in mice. Cell Cycle.

[60] Liu L, Wang G, Wang L, Yu C, Li M, Song S, Hao L, Ma L, Zhang Z (2020). Computational identification and characterization of glioma candidate biomarkers through multi-omics integrative profiling. Biol Direct.

[61] Chierici M, Francescatto M, Bussola N, Jurman G, Furlanello C (2020). Predictability of drug-induced liver injury by machine learning. Biol Direct.

[62] Gallo M, Paludi D, Cicero DO, Chiovitti K, Millo E, Salis A, Damonte G, Corsaro A, Thellung S, Schettini G (2005). Identification of a conserved N-capping box important for the structural autonomy of the prion alpha 3-helix: the disease associated D202N mutation destabilizes the helical conformation. Int J Immunopathol Pharmacol.

[63] Han Y, Ye X, Wang C, Liu Y, Zhang S, Feng W, Huang K, Zhang J (2019). Integration of molecular features with clinical information for predicting outcomes for neuroblastoma patients. Biol Direct.

[64] Han Y, Ye X, Cheng J, Zhang S, Feng W, Han Z, Zhang J, Huang K (2019). Integrative analysis based on survival associated co-expression gene modules for predicting Neuroblastoma patients' survival time. Biol Direct.

[65] Dao T, Pankov D, Scott A, Korontsvit T, Zakhaleva V, Xu Y, Xiang J, Yan S, de Morais Guerreiro MD, Veomett N (2015). Therapeutic bispecific T-cell engager antibody targeting the intracellular oncoprotein WT1. Nat Biotechnol.

[66] Fesnak AD, June CH, Levine BL (2016). Engineered T cells: the promise and challenges of cancer immunotherapy. Nat Rev Cancer.

[67] Chowdhury S, Beitel LK, Lumbroso R, Purisima EO, Paliouras M, Trifiro M (2019). A targeted bivalent androgen receptor binding compound for prostate cancer therapy. Horm Cancer.

[68] Lim SM, Pyo KH, Soo RA, Cho BC (2021). The promise of bispecific antibodies: Clinical applications and challenges. Cancer Treat Rev.

[69] MacDonald L, Jenkins J, Purvis G, Lee J, Franco AT (2020). The thyroid tumor microenvironment: potential targets for therapeutic intervention and prognostication. Horm Cancer.

[70] Garrido F (2019). HLA Class-I expression and cancer immunotherapy. Adv Exp Med Biol.

[71] Hou AJ, Chen LC, Chen YY (2021). Navigating CAR-T cells through the solid-tumour microenvironment. Nat Rev Drug Discov.

[72] DePeaux K, Delgoffe GM (2021). Metabolic barriers to cancer immunotherapy. Nat Rev Immunol.

[73] Wang L, Liu Y, Du T, Yang H, Lei L, Guo M, Ding HF, Zhang J, Wang H, Chen X (2020). ATF3 promotes erastin-induced ferroptosis by suppressing system Xc. Cell Death Differ.

[74] Vance JE (2020). Inter-organelle membrane contact sites: implications for lipid metabolism. Biol Direct.

[75] Casimiro-Soriguer CS, Loucera C, Perez Florido J, Lopez-Lopez D, Dopazo J (2019). Antibiotic resistance and metabolic profiles as functional biomarkers that accurately predict the geographic origin of city metagenomics samples. Biol Direct.

[76] Lee PMY, Kwok CH, Chan WC, Wu C, Tsang KH, Law SH, Yeung YC, Wang F, Yang XR, Tse LA (2020). Heterogeneous associations between obesity and reproductive-related factors and specific breast cancer subtypes among hong kong chinese women. Horm Cancer.

[77] Licht JD, Bennett RL (2021). Leveraging epigenetics to enhance the efficacy of immunotherapy. Clin Epigenetics.

[78] Sumsion GR, Bradshaw MS, Beales JT, Ford E, Caryotakis GRG, Garrett DJ, LeBaron ED, Nwosu IO, Piccolo SR (2020). Diverse approaches to predicting drug-induced liver injury using gene-expression profiles. Biol Direct.

[79] Li KP, Ladle BH, Kurtulus S, Sholl A, Shanmuganad S, Hildeman DA (2020). T-cell receptor signal strength and epigenetic control of Bim predict memory CD8(+) T-cell fate. Cell Death Differ.

[80] Jing YY, Cai FF, Zhang L, Han J, Yang L, Tang F, Li YB, Chang JF, Sun F, Yang XM (2020). Epigenetic regulation of the Warburg effect by H2B monoubiquitination. Cell Death Differ.

[81] Nazio F, Bordi M, Cianfanelli V, Locatelli F, Cecconi F (2019). Autophagy and cancer stem cells: molecular mechanisms and therapeutic applications. Cell Death Differ.

[82] Maiuri MC, Kroemer G (2019). Therapeutic modulation of autophagy: which disease comes first?. Cell Death Differ.

[83] Germic N, Frangez Z, Yousefi S, Simon HU (2019). Regulation of the innate immune system by autophagy: monocytes, macrophages, dendritic cells and antigen presentation. Cell Death Differ.

[84] Germic N, Frangez Z, Yousefi S, Simon HU (2019). Regulation of the innate immune system by autophagy: neutrophils, eosinophils, mast cells NK cells. Cell Death Differ.

[85] Seton-Rogers S (2019). Eliminating protective autophagy in KRAS-mutant cancers. Nat Rev Cancer.

[86] Willson J (2020). Mitosis flips the switch on autophagy control. Nat Rev Mol Cell Biol.

[87] Ozten N, Vega K, Liehr J, Huang X, Horton L, Cavalieri EL, Rogan EG, Bosland MC (2019). Role of estrogen in androgen-induced prostate carcinogenesis in NBL rats. Horm Cancer.

[88] Fowler AM, Salem K, DeGrave M, Ong IM, Rassman S, Powers GL, Kumar M, Michel CJ, Mahajan AM (2020). Progesterone receptor gene variants in metastatic estrogen receptor positive breast cancer. Horm Cancer.

[89] Del Moral-Morales A, Gonzalez-Orozco JC, Capetillo-Velazquez JM, Pina-Medina AG, Camacho-Arroyo I (2020). The role of mPRdelta and mPRepsilon in human glioblastoma cells: expression, hormonal regulation, and possible clinical outcome. Horm Cancer.

[90] Li X, Zhang H, Zhou Y, Cheng R (2021). Risk factors for central lymph node metastasis in the cervical region in papillary thyroid carcinoma: a retrospective study. World J Surg Oncol.

[91] Santoro A, Mele E, Marino M, Viggiano A, Nori SL, Meccariello R (2021). The complex interplay between endocannabinoid system and the estrogen system in central nervous system and periphery. Int J Mol Sci.

[92] Davis PJ, Mousa SA, Schechter GP, Lin HY (2020). Platelet ATP, thyroid hormone receptor on integrin alphavbeta3 and cancer metastasis. Horm Cancer.

[93] Sepich-Poore GD, Zitvogel L, Straussman R, Hasty J, Wargo JA, Knight R (2021). The microbiome and human cancer. Science.

[94] Lennon JT, Locey KJ (2020). More support for Earth's massive microbiome. Biol Direct.

[95] Zhu C, Miller M, Lusskin N, Mahlich Y, Wang Y, Zeng Z, Bromberg Y (2019). Fingerprinting cities: differentiating subway microbiome functionality. Biol Direct.

[96] Eshhar Z, Waks T, Gross G, Schindler DG (1993). Specific activation and targeting of cytotoxic lymphocytes through chimeric single chains consisting of antibody-binding domains and the gamma or zeta subunits of the immunoglobulin and T-cell receptors. Proc Natl Acad Sci USA.

[97] Gross G, Waks T, Eshhar Z (1989). Expression of immunoglobulin-T-cell receptor chimeric molecules as functional receptors with antibody-type specificity. Proc Natl Acad Sci USA.

[98] D'Aloia MM, Zizzari IG, Sacchetti B, Pierelli L, Alimandi M (2018). CAR-T cells: the long and winding road to solid tumors. Cell Death Dis.

[99] Melero I, Castanon E, Alvarez M, Champiat S, Marabelle A (2021). Intratumoural administration and tumour tissue targeting of cancer immunotherapies. Nat Rev Clin Oncol.

[100] Morris EC, Neelapu SS, Giavridis T, Sadelain M (2021). Cytokine release syndrome and associated neurotoxicity in cancer immunotherapy. Nat Rev Immunol.

[101] Zhang Z, Jiang D, Yang H, He Z, Liu X, Qin W, Li L, Wang C, Li Y, Li H (2019). Modified CAR T cells targeting membrane-proximal epitope of mesothelin enhances the antitumor function against large solid tumor. Cell Death Dis.

[102] Nagarsheth NB, Norberg SM, Sinkoe AL, Adhikary S, Meyer TJ, Lack JB, Warner AC, Schweitzer C, Doran SL, Korrapati S (2021). TCR-engineered T cells targeting E7 for patients with metastatic HPV-associated epithelial cancers. Nat Med.

[103] Murata K, Nakatsugawa M, Rahman MA, Nguyen LT, Millar DG, Mulder DT, Sugata K, Saijo H, Matsunaga Y, Kagoya Y (2020). Landscape mapping of shared antigenic epitopes and their cognate TCRs of tumor-infiltrating T lymphocytes in melanoma. Elife.

[104] Sugata K, Matsunaga Y, Yamashita Y, Nakatsugawa M, Guo T, Halabelian L, Ohashi Y, Saso K, Rahman MA, Anczurowski M (2021). Affinity-matured HLA class II dimers for robust staining of antigen-specific CD4(+) T cells. Nat Biotechnol.

[105] Rahman MA, Murata K, Burt BD, Hirano N (2021). Changing the landscape of tumor immunology: novel tools to examine T cell specificity. Curr Opin Immunol.

[106] Qu Q, Li Y, Fang X, Zhang L, Xue C, Ge X, Wang X, Jiang Y (2019). Differentially expressed tRFs in CD5 positive relapsed & refractory diffuse large B cell lymphoma and the bioinformatic analysis for their potential clinical use. Biol Direct.

[107] Ryan FJ (2019). Application of machine learning techniques for creating urban microbial fingerprints. Biol Direct.

[108] Harris ZN, Dhungel E, Mosior M, Ahn TH (2019). Massive metagenomic data analysis using abundance-based machine learning. Biol Direct.

